# Low Temperature Affects Fatty Acids Profiling and Key Synthesis Genes Expression Patterns in *Zanthoxylum bungeanum* Maxim

**DOI:** 10.3390/ijms23042319

**Published:** 2022-02-19

**Authors:** Jieyun Tian, Lu Tian, Ming Chen, Yabing Chen, Anzhi Wei

**Affiliations:** 1 College of Forestry, Northwest A&F University, Xianyang 712100, China; tianjieyun@nwafu.edu.cn (J.T.); t1anlu@nwafu.edu.cn (L.T.); 15511031817@nwafu.edu.cn (M.C.); 2019055403@nwafu.edu.cn (Y.C.); 2 Research Centre for Engineering and Technology of Zanthoxylum, State Forestry Administration, Xianyang 712100, China

**Keywords:** *Zanthoxylum bungeanum*, cold stress, fatty acid profiling, chemometrics analysis, key genes

## Abstract

*Zanthoxylum bungeanum* is one of the most important medicinal and edible homologous plants because of its potential health benefits and unique flavors. The chemical components in compositions and contents vary with plant genotype variations and various environmental stress conditions. Fatty acids participate in various important metabolic pathways in organisms to resist biotic and abiotic stresses. To determine the variations in metabolic profiling and genotypes, the fatty acid profiling and key differential genes under low temperature stress in two *Z. bungeanum* varieties, cold-tolerant (FG) and sensitive (FX), were investigated. Twelve main fatty acids were found in two *Z. bungeanum* varieties under cold stress. Results showed that the contents of total fatty acids and unsaturated fatty acids in FG were higher than those in FX, which made FG more resistant to low temperature. Based on the result of orthogonal partial least squares discriminant analysis, palmitic acid, isostearic acid, linolenic acid and eicosenoic acid were the important differential fatty acids in FG under cold stress, while isomyristic acid, palmitic acid, isostearic acid, stearic acid, oleic acid, linolenic acid and eicosenoic acid were the important differential fatty acids in FX. Furthermore, fatty acid synthesis pathway genes fatty acyl-ACP thioesterase A (*FATA*), Delta (8)-fatty-acid desaturase 2 (*SLD2*), protein ECERIFERUM 3 (*CER3*), fatty acid desaturase 3 (*FAD3*) and fatty acid desaturase 5 (*FAD5*) played key roles in FG, and *SLD2*, *FAD5*, 3-oxoacyl-[acyl-carrier-protein] synthase I (*KAS I*), fatty acyl-ACP thioesterase B (*FATB*) and acetyl-CoA carboxylase (*ACC*) were the key genes responding to low temperature in FX. The variation and strategies of fatty acids in two varieties of *Z. bungeanum* were revealed at the metabolic and molecular level. This work provides a reference for the study of chemical components in plant stress resistance.

## 1. Introduction

*Zanthoxylum* is a tree genus of the Rutaceae family, which is widely cultivated in China, Japan, Korea and other eastern Asian countries [1]. *Zanthoxylum bungeanum* is popular in temperate and subtropical regions of China. The pericarps of *Z. bungeanum* are rich in alkamides and possess a unique numbing taste characteristic, which is indispensable in Sichuan hot pot and other foods [2]. The *Z. bungeanum* pericarps contain a lot of volatile oil, which can be extracted to make cosmetics. The sprouts of *Z. bungeanum* can be eaten in vegetable salads. In addition, the fruits and leaves can also be used as traditional Chinese medicine, with therapeutic anti-inflammatory effects as well as dispelling wind and dampness and strengthening the stomach [3,4,5]. *Z. bungeanum* is the dominating species widely planted in China. The *Z. bungeanum* tree likes light and warm temperatures, and needs higher temperatures during its growth and development. In spring, *Z. bungeanum* often suffers from late spring cold, which seriously affects its growth and development. Thus, temperature is an important limiting factor affecting the natural distribution and yield of *Z. bungeanum*. In addition, with the widespread instability of global climate change and the frequency of extreme weather, the research of plant stress resistance is becoming more urgent. Therefore, it is of great significance and value to study the response mechanism of *Z. bungeanum* to low temperature.

Fatty acids are the major components in oil crop seeds, which store the energy produced by photosynthesis in the form of storage lipids in plants [6]. In other plants or tissues, fatty acids are essential for cellular metabolism and biology [7]. Phospholipids constitute the bilayer of the biological membranes. Phospholipids are lipids containing phosphoric acid. Two hydrocarbon chains of fatty acids form the fat-soluble hydrophobic tail of phospholipids, which create membrane fluidity. Fatty acids can also function as signaling lipids in signaling pathways [8]. Furthermore, fatty acids occur in the defense response of plants to biotic and abiotic stresses [9]. Temperature is a variable abiotic environmental factor that affects the growth of plants. When plants are subjected to low temperature stress, biomembranes are the first to be harmed. The fluidity and stability of membranes are closely related to plant cold resistance. Unsaturated fatty acids are generally considered to be beneficial for maintaining the stability of membranes [10,11]. Unsaturated fatty acids contain one or more double bonds, which make it difficult for molecules to pack tightly, thus increasing the fluidity of the membranes. The increased fluidity of the membrane can maintain liquid form under low temperature stress, so the stability is increased and the cold resistance of the plant is enhanced. Linolenic acid (C18:3) and hexadecatrienoic acid (C16:3) have been found to maintain the fluidity and stability of the chloroplast membrane under cold stress [12]. Therefore, increasing the contents of unsaturated fatty acids in membrane lipids can enhance cold resistance in low temperature environments [13]. The unsaturation of membrane lipids is positively correlated with the activity of fatty acid desaturase. When the activity of fatty acid desaturase increases, the membrane lipid unsaturation and fluidity increase, and the stability of the plant membrane system is improved at low temperatures [14,15]. The enzymes and related genes in fatty acid synthesis pathway have been reported to respond to low temperature [16,17]. In addition, very long-chain fatty acids and derivatives are the components of cutin and wax in plant surfaces, and they constitute a barrier for the isolation of plant cells from the environment [18]. For cold-sensitive plants, low temperature stress will first damage the cells, resulting in a freezing phenomenon between plant cells. Under low temperature stress, a large number of horizontal lamellar cuticle crystals will be produced in the plant epidermis. These lamellar crystal structures can act as a cold barrier to some extent, thus reducing the risk of cell freezing [19,20,21].

The effects of fatty acids on plant resistance have been paid much attention, and the fatty acid response mechanism to low temperature has been reported by many studies. Several genes involved in the fatty acid synthesis play critical roles in plant cold tolerance. Overexpression of *Linum usitatissimum FAD2A* and *FAD3A* in *Arabidopsis thaliana* seeds leads to an increase in linolenic acid content, which promotes jasmonic acid synthesis and enhances plant cold tolerance [22]. Similar results were obtained in Rice [13]. In addition, it has been reported that MYB transcription factor can modulate fatty acid biosynthesis by repressing the expression of *MaFAD3* at transcriptional level under cold stress in banana fruit [23]. A circadian clock-related gene, *TIC*, a target of R2R3 transcription factor, regulates plant cold tolerance by promoting unsaturation of fatty acids [24]. The findings imply that the response of plant fatty acids to low temperature in plants does not exist independently, but acts by participating in the cold response network.

Due to the uncontrollability of temperature, research on the cold responses of plants lags behind that of other stresses, such as drought, and research on the stress resistance of *Z. bungeanum* is also relatively slow. The difference of fatty acid composition and its variation among *Z. bungeanum* varieties has not been reported. In this work, we selected two *Z. bungeanum* varieties with different cold resistance, and studied the differences and changes in fatty acid components under low temperature stress. Chemometrics analysis was employed to screen the important fatty acids of cold-tolerant and cold-sensitive varieties under cold stress. Furthermore, the key genes responding to low temperature in the fatty acid synthesis pathway, which play a decisive role in the changes in fatty acid composition in *Z. bungeanum,* were identified. This work provides a train of thought for the study of chemical components in the stress resistance of *Z. bungeanum*, and provides a reference and basis for the cultivation of high-quality and high-yield varieties of *Z. bungeanum*. Furthermore, it provides a reference for the research topic of plant low-temperature response mechanisms.

## 2. Results

### 2.1. Fatty Acid Profiling in Leaves under 4 °C Cold Stress for Different Time Treatments among Two Z. bungeanum Varieties

**Figure 1:**
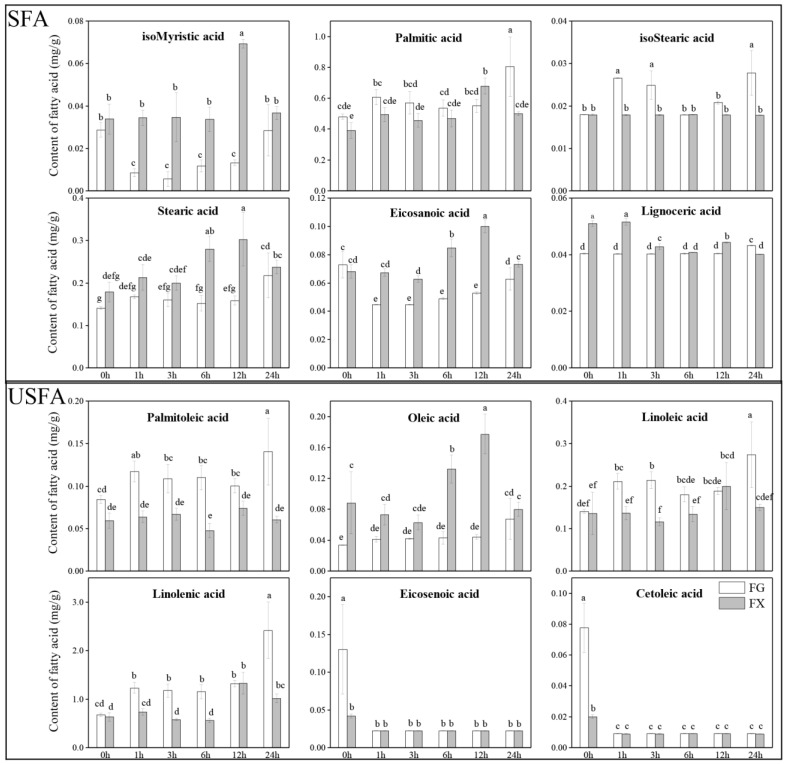
Fatty acid profiling of FG and FX leaf samples under 4 °C low-temperature stress for 24 h. (**SFA**): saturated fatty acid; (**USFA**): unsaturated fatty acid. The data bars represent the mean value of 3 samples taken at each time point, and the error bars represent the standard deviation. The letters above the data bars mean significance at *p* < 0.05.

Twelve fatty acids in the leaves of cold-tolerant and sensitive *Z. bungeanum* varieties were detected: isomyristic acid (0.03 mg·g^−1^ in FG, 0.03 mg·g^−1^ in FX), palmitic acid (0.48, 0.39 mg·g^−1^), palmitoleic acid (0.08, 0.06 mg·g^−1^), isostearic acid (0.02, 0.02 mg·g^−1^), stearic acid (0.14, 0.18 mg·g^−1^), oleic acid (0.03, 0.09 mg·g^−1^), linoleic acid (0.14, 0.14 mg·g^−1^), linolenic acid (0.67, 0.63 mg·g^−1^), eicosanoic acid (0.07, 0.07 mg·g^−1^), eicosenoic acid (0.13, 0.04 mg·g^−1^), cetoleic acid (0.08, 0.02 mg·g^−1^) and lignoceric acid (0.04, 0.05 mg·g^−1^) (Figure 1). Under normal growth conditions, the contents of eicosenoic acid and cetoleic acid in FG were significantly higher than in FX, while the contents of oleic acid and lignoceric acid were significantly lower in FG than in FX. There was no significant difference in the content of the remaining eight fatty acids between FG and FX. Notably, palmitic acid was the major saturated fatty acid (SFA), and linolenic acid was the dominating unsaturated fatty acid (USFA) in *Z. bungeanum,* whether the leaves experienced low-temperature stress or not. For the cold-tolerant variety FG, the content of palmitic acid and linolenic acid showed same increasing trends and reached the peaks of 0.80 and 2.41 mg·g^−1^ within 24 h of cold stress. For the cold-sensitive variety FX, significant increases in the content of palmitic acid and linolenic acid were found at 12 h upon cold treatment, which showed the same trends of first increasing and then decreasing during 24 h of the low-temperature stress process. In FG, the contents of palmitic acid, palmitoleic acid, isostearic acid and linoleic acid were significantly high at 24 h, while the contents of isomyristic acid, stearic acid, oleic acid and eicosanoic acid were significantly high at 12 h. The total fatty acid (TFA) content in FG at 24 h was significantly higher than that at other time points, while the TFA content in FX was highest at 12 h (Table 1). The USFAs were the main fatty acids in all samples studied, and the change trend in USFAs with the duration of cold-stress treatment was consistent with that of TFAs in two varieties. In general, the contents of TFAs and USFAs in FG were higher than those in FX. In FG, the index of unsaturated fatty acids (IUFA) in samples under low-temperature stress was significantly higher than that in control. In FX, IUFA increased significantly only in samples treated for 12 h.

### 2.2. Chemometric Analysis of Fatty Acid Components in Z. bungeanum

#### 2.2.1. Cluster Heat Map Analysis

**Figure 2:**
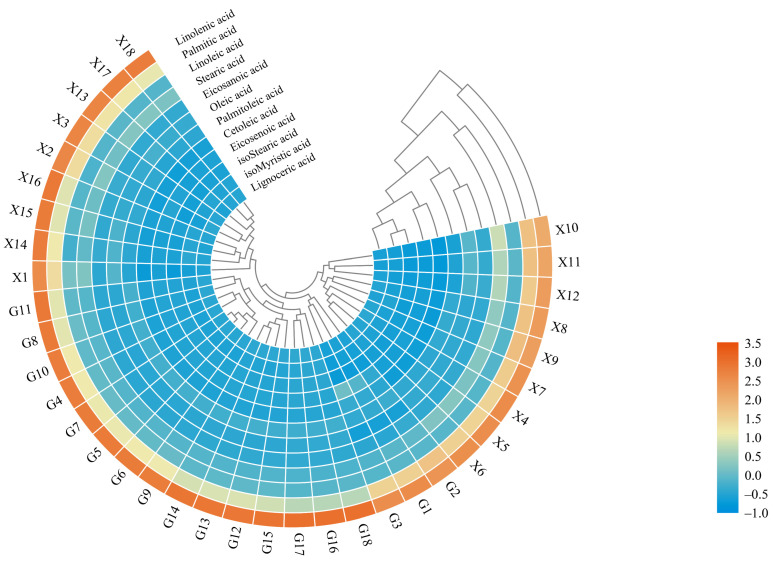
Cluster heat map (CHM) analysis of fatty acid composition for two varieties of FG and FX samples under 4 °C cold stress. G denotes the cold-tolerant cultivar “Fuguhuajiao” and X denotes the cold-sensitive cultivar “Fengxiandahongpao”.

Cluster heat map (CHM) was applied to classify the 36 leaf samples at different time points of two *Z. bungeanum* varieties based on the normalized values of fatty acid composition. In addition, the fatty acid composition profiling from different cold-stress treated samples were also observed. Linolenic acid and palmitic acid shared a similar trend in the leaf samples, while linoleic acid and stearic acid also shared a similar trend, and the remaining eight components shared another similar trend. A similar fatty acid trend means a similar accumulation and response profiling under cold stress. The samples with different treatment times in two varieties were divided into four groups: the first group contained FX1–3 (0 h), FX13–15 (12 h) and FX16–18 (24 h); the second group contained FG4–6 (1 h), FG7–9 (3 h), FG10–12 (6 h), FG13–15 (12 h) and FG 16–18 (24 h); the third group contained FG1–3 (0 h); the fourth group contained FX4–6 (1 h), FX7–9 (3 h) and FX10–12 (6 h) (Figure 2).

#### 2.2.2. Principal Component Analysis

**Figure 3:**
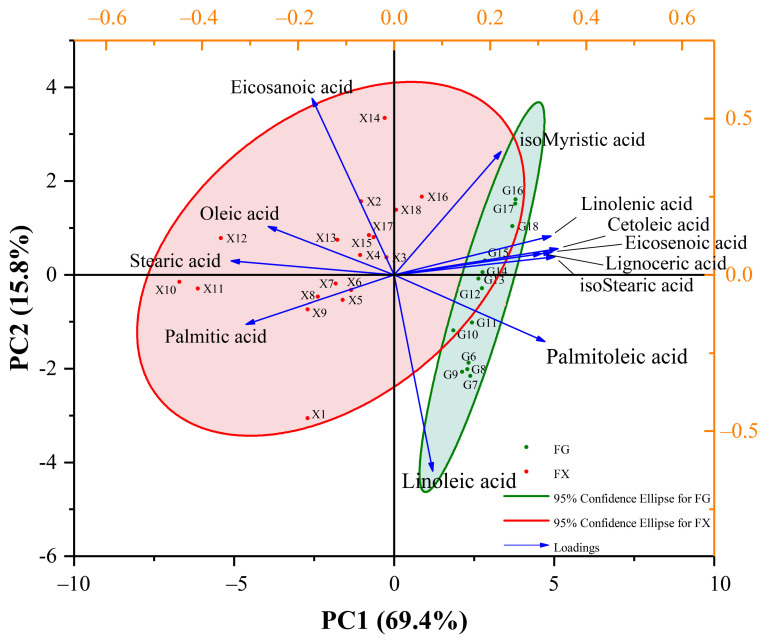
Principal component analysis (PCA) of fatty acid composition for FG and FX samples under 4 °C cold stress.

Principal component analysis (PCA) was carried out to better understand the chemometric characteristics of the fatty acid composition under different cold-stress treatment durations between cold-tolerant and cold-sensitive varieties. PCA clearly separated the two varieties according to their cold-stress treatment. The first two principal components (PCs) were PC1 (69.4%) and PC2 (15.8%) of the total variances, which could explain the variation in the fatty acid components in two varieties of *Z. bungeanum*. PC1 had high component loadings of cetoleic acid and stearic acid, which were the prominent factors causing the differences between two varieties. Furthermore, PC2 had high component loadings from eicosanoic acid and linoleic acid, indicating that they were the main composition contributors to the classification of samples treated with different stress durations in each variety (Figure 3). Taken together, there were differences in the fatty-acid response strategies of cold-tolerant and cold-sensitive varieties under low-temperature stress.

#### 2.2.3. Orthogonal Partial Least Squares Discriminant Analysis (OPLS-DA)

**Figure 4:**
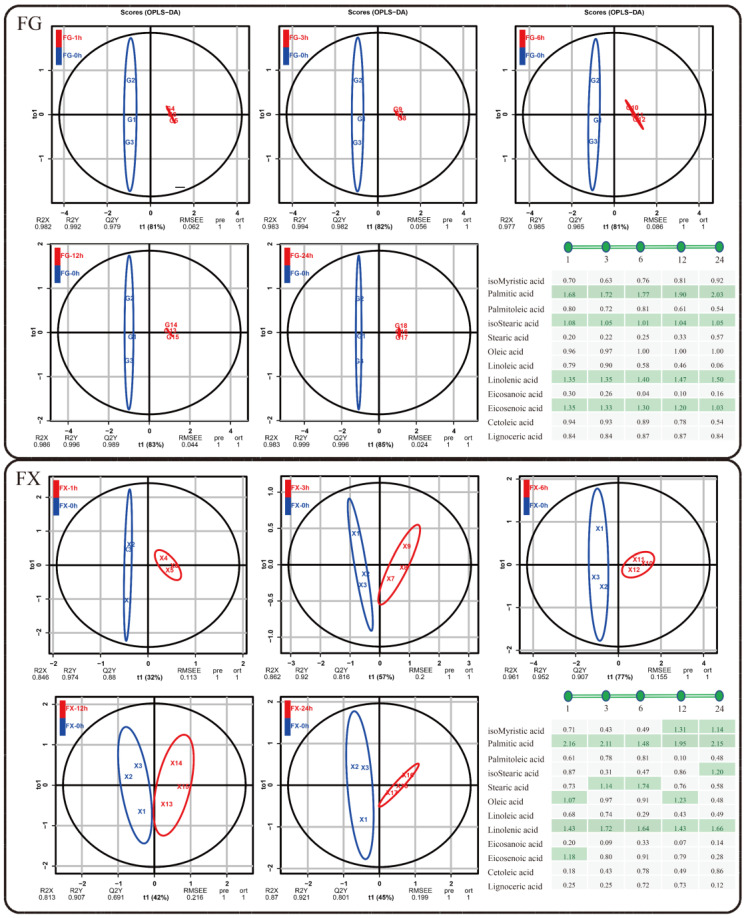
Orthogonal partial least squares discriminant analysis (OPLS-DA) and variable importance in projection (VIP) of fatty acid composition for (**FG**) and (**FX**) samples under low temperature.

OPLS-DA chemometric method was employed to screen differential fatty acids in samples among different low-temperature stress times. Additionally, variable importance in projection (VIP) threshold was introduced to measure the influence intensity of different components in cold-stress treatment samples. The OPLS-DA models were constructed by FG1–3 (0 h) vs. FG4–6 (1 h), FG1–3 (0 h) vs. FG7–9 (3 h), FG1–3 (0 h) vs. FG10–12 (6 h), FG1–3 (0 h) vs. FG13–15 (12 h), and FG1–3 (0 h) vs. FG16–18 (24 h), and the same in FX. OPLS-DA plot showed a clear separation of two varieties (Figure 4). In this study, fatty acid components with VIP > 1 were considered to have strong influence and explanatory power on the cold-stress treatment samples. The VIP values of palmitic acid, isostearic acid, linolenic acid and eicosenoic acid were more than one, indicating that these fatty acid compositions were important metabolites at each cold-stress time point (1 h, 3 h, 6 h, 12 h, 24 h) comparing with at 0 h in FG. In FX, palmitic acid and linolenic acid played crucial roles as important metabolites in the whole process of low-temperature stress. Other compositions, such as isomyristic acid, isostearic acid and oleic acidfunctioned at late stages (12 h, 24 h) of cold stress, while stearic acid, oleic acid, and eicosenoic acid were important fatty acid metabolites at early stages (1 h, 3 h, 6 h) of cold stress.

### 2.3. Correlation and Redundancy (RDA) Analysis of Fatty Acid Content and Synthesis-Related Genes

**Figure 5:**
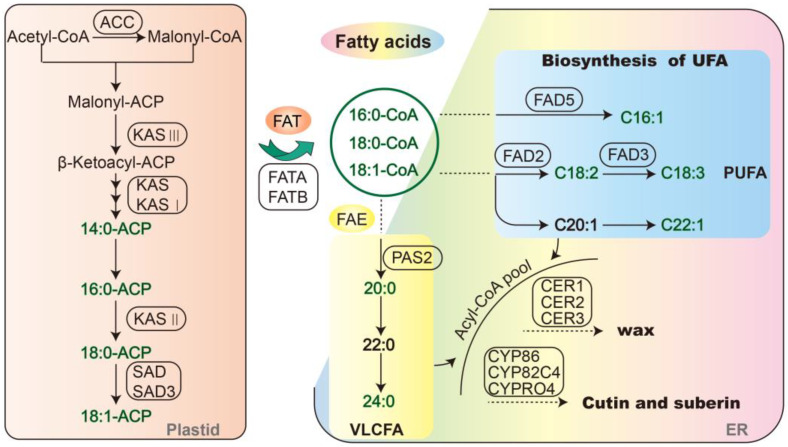
Fatty acid biosynthesis pathway and main related genes. ACC, acetyl-CoA carboxylase; KAS, 3-ketoacyl-ACP synthase; SAD, stearoyl-ACP desaturase; FAD, fatty acid desaturase; FAT, fatty acyl-ACP thioesterase; FAE, fatty acid elongase complex; PAS, very-long-chain (3R)-3-hydroxyacyl-CoA dehydratase PASTICCINO 2; CER, ECERIFERUM; CYP, Cytochrome P450; UFA, unsaturated fatty acids; PUFA, polyunsaturated fatty acids; VLCFA, very long-chain fatty acids; ER, endoplasmic reticulum.

The level of fatty acid content is regulated by genes in the fatty acid synthesis pathway. In order to investigate which fatty acid synthesis-related genes play key roles under low-temperature stress in two varieties, correlation coefficient and RDA methods were used for analysis. According to the KEGG functional annotation of transcriptome sequencing and previous reports, 20 genes involved in the key steps of the fatty acid synthesis pathway were selected for expression pattern analysis. These 20 fatty acid-related genes were involved in the fatty acid synthesis, the fatty acid desaturation and the fatty acid elongation (Figure 5).

The results of correlation analysis in FG showed that the important metabolite palmitic acid had a positive correlation of 0.83 with *FATA*, and had negative correlation of 0.85 and 0.83 with *CER3* and *SLD2*, respectively. Similarly, stearic acid has a high positive correlation of 0.87 with gene *FATA*, and a negative correlation of 0.82 and 0.97 with *CER3* and *SLD2*, respectively. The unsaturated fatty acid linolenic acid was positively correlated with *CER3* and *SLD2* by 0.82 and 0.76, and negatively correlated with *FATA* by 0.80. The long chain USFA eicosenoic acid was positively correlated with *FAD3* and negatively correlated with *SAD3*. Among the seven important metabolites of FX, stearic acid was positively correlated with *SLD2*, *KAS I* and *FATB* by 0.80, 0.72 and 0.67, respectively. The USFA oleic acid had a high positive correlation with a series of genes, namely, *SLD2* (0.85), *KAS I* (0.80), *KAS* (0.74), *SAD* (0.71), *KAS III* (0.70), *FAD2* (0.70), *FAD5* (0.70), *CER3* (0.69) and *FATB* (0.68). Meanwhile, eicosenoic acid had a high negative correlation with *KAS I*, *FATB* and *SLD2* (Figure S1).

**Figure 6:**
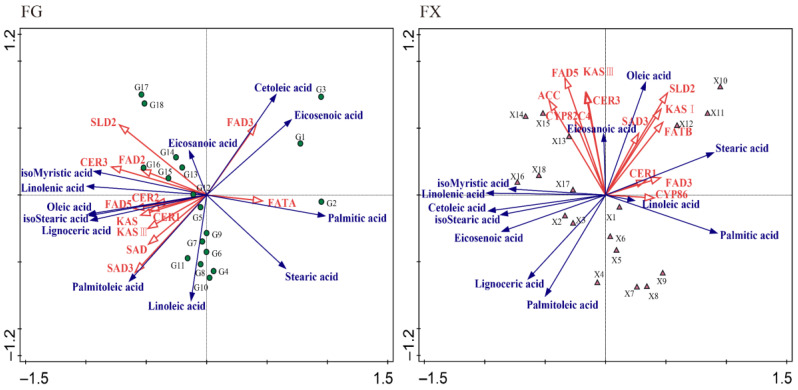
Redundancy analysis (RDA) of fatty acid composition for (**FG**) and (**FX**) samples under low temperature.

RDA can identify the prominent factors that have direct or indirect influence on variables. We used RDA to identify the main fatty acid synthesis-related genes that have important influence on fatty acid compositions under low-temperature stress. In the RDA result figure, the composition variables are vertically projected onto gene variable rays, and the larger the value of this intersection point on the coordinate axis, the greater the influence. The longer arrow length, the greater influence. In addition, the smaller the angle between the rays, the higher the correlation. In this way, we can find the genes that have great influence on each fatty acid composition. The important fatty acid metabolites in the two varieties were obtained through OPLS-DA analysis, therefore, we mainly focused on the genes that have important influence on these fatty acids. RDA results showed that gene *FATA* had an important influence on palmitic acid, *FAD5* had an important influence on isoStearic acid, *SLD2* and *CER3* had important influences on linolenic acid and *FAD3* had an important influence on eicosenoic acid in FG. In FX, genes *FAD5* and *ACC* contributed to isoMyristic acid and linolenic acid and *SLD2*, *KAS I* and *FATB* contributed to stearic acid and oleic acid (Figure 6). In general, *FATA*, *FAD5*, *SLD2*, *CER3* and *FAD3* were identified as playing important roles in the fatty acid synthesis pathway in FG under cold stress, while the key genes in FX were *FAD5*, *ACC*, *SLD2*, *KAS I* and *FATB*.

### 2.4. Weighted Gene Co-Expression Network Analysis (WGCNA) of Fatty Acids and Transcriptome in Response to Low Temperature in Z. bungeanum

**Figure 7:**
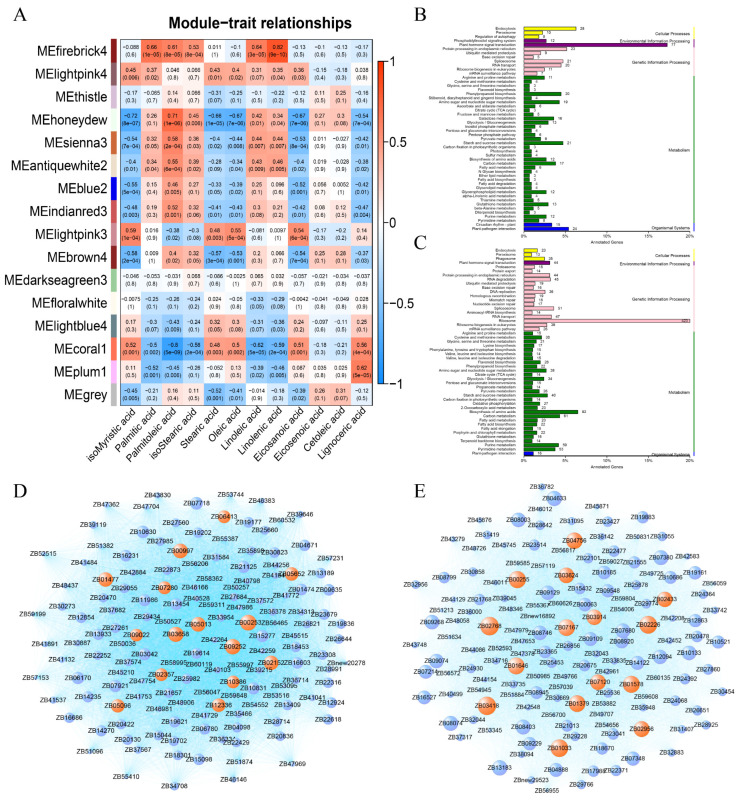
Weighted gene co-expression network analysis (WGCNA) for fatty acids in *Z. bungeanum*. (**A**) Correlations between modules and fatty acid composition traits; (**B**) KEGG annotation of genes in MEfirebrick4 module; (**C**) KEGG annotation of genes in MElightpink3 module; (**D**) Co-expression network of genes in MEfirebrick4 module; (**E**) Co-expression network of genes in MElightpink3 module.

The fatty acids-related gene network was constructed by combining the fatty acid contents with the transcriptome profile by WGCNA. After optimizing and merging the imported data, 16 trait-related modules were finally obtained (Figure 7A). The results showed that MEfirebrick4 module had high correlation with palmitic acid, isostearic acid and linolenic acid, which were the important metabolisms in FG. MElightpink3 module had high correlation with isomyristic acid, stearic acid and oleic acid, which were the important metabolisms in FX. Thus, we selected MEfirebrick4 and MElightpink3 modules for further network construction and analysis. Functional annotation analysis of the genes in the two modules was performed. The gene network was constructed using the top 150 core genes in each module (Figure 7D,E).

In MEfirebrick4 module (Figure 7B), 77 genes were enriched in the plant hormone signal transduction pathway (ko04075), 28 genes were enriched in endocytosis (ko04144), 23 genes were enriched in protein processing in endoplasmic reticulum (ko04141), 15 genes were enriched in plant circadian rhythm (ko04712) and 12 genes were enriched in phosphatidylinositol signaling system (ko04070). In this module, fatty acid synthesis-related genes were enriched in glycerophospholipid metabolism (ko00564), fatty acid metabolism (ko01212), alpha-Linolenic acid metabolism (ko00592), glycerolipid metabolism (ko00561), glycosphingolipid biosynthesis (ko00603), cutin, suberine and wax biosynthesis (ko00073) and other related pathways. In MElightpink3 module (Figure 7C), 92 genes were enriched in biosynthesis of amino acids (ko01230), 44 genes were enriched in protein processing in endoplasmic reticulum (ko04141), 44 genes were enriched in plant hormone signal transduction (ko04075), 28 genes in flavonoid biosynthesis (ko00941) and 3 genes in ABC transporters (ko02010). In addition, we found that many genes in this module were enriched in sugar-related pathways, such as starch and sucrose metabolism (ko00500), amino sugar and nucleotide sugar metabolism (ko03008) and fructose and mannose metabolism (ko00051).

## 3. Discussion

The present work showed that low temperature influenced the fatty acid compositions and contents of *Z. bungeanum*. In all samples of this study, palmitic acid and linolenic acid were the most abundant fatty acids in *Z. bungeanum* leaves. One of the reasons may be that the two varieties of *Z. bungeanum* selected have more of these two fatty acids, which is determined by the genetic characteristics of this plant. The cold resistance of *Z. bungeanum* is a kind of acquired ability gradually developed and formed in the process of long-term adaptation to low-temperature stress. On the other hand, studies have shown that the proportion of USFA in plants increases after plants are subjected to low-temperature stress [25,26]. Moreover, C16/C18 fatty acids are the primary derivative source of the tail of phospholipids, which is the main framework of biomembrane lipid bilayer [27]. This indicated that in the process of cold response, *Z. bungeanum* supplemented the structural components of cell membrane by increasing the content of palmitic acid and linolenic acid, and maintained the membrane fluidity by synthesizing more unsaturated fatty acids. In addition, the total amount of fatty acids and the content of unsaturated fatty acids in cold-tolerant variety FG were higher than those in cold-sensitive variety FX. Furthermore, the change trend in fatty acids varied in the two varieties, increasing from 0 h to 24 h in FG, and increasing from 0 h to 12 h then decreasing in FX. When plants are subjected to low-temperature stress, the cell membrane system undergoes phase transformation and change from the liquid crystalline phase to the gel phase. Under cold stress, the membrane lipid phase transition temperature of cold-tolerant plants is lower than that of cold-sensitive plants. Lyons [28] believed that this is mainly because the unsaturated fatty acid content in the membrane lipid of cold-tolerant plants is higher than that of cold-sensitive plants. Therefore, the high content of total fatty acids and unsaturated fatty acids in FG is one of the reasons why the cold resistance of FG is higher than that of FX. Furthermore, OPLS-DA results showed that palmitic acid, isostearic acid, linolenic acid and eicosenoic acid were important indicator metabolites in FG samples. Based on this, we infer that these four fatty acids help to improve the cold resistance of FG.

Chemometrics methods can successfully classify samples according to the characteristics of the metabolites. The CHM clustering results showed that changes in fatty acid composition in low-temperature treated samples affected the growth and cold resistance of two varieties. The samples from 1 h to 24 h in FG were divided into a group indicating that the fatty acid response pathway in plants was activated once it received the cold signal and could continue to resist the adversity. However, the 12 h and 24 h samples in FX were clustered with 0 h samples, which indicated that the response time of FX to low temperature was longer than that of FG, and it took 12 h for the stress response to reach the state of fatty acid pathway in the control. PCA results showed that the different fatty acid compositions clearly separated the samples of the two varieties after low-temperature stress. These variables can be used as characteristic chemical peaks to distinguish different samples of *Z. bungeanum* under cold-stress response. OPLS-DA was used to screen and identify the important fatty acid metabolites of two varieties under low-temperature stress. The important fatty acid differences in two varieties were not consistent. These results indicate that the changes in fatty acid composition and coping strategies vary in different *Z. bungeanum* varieties in response to low-temperature stress. This conclusion is consistent with previous works of Ma [29] and Cruz [30].

Fatty acid biosynthesis pathway includes de novo synthesis, fatty acid desaturation and fatty acid elongation (Figure 5). De novo synthesis of fatty acids in plants occurs in plastids. The starting material of the reaction is Acety1-CoA. Acetyl-CoA is catalyzed by acetyl-CoA carboxylase (ACC) to malonyl-CoA. Under the catalysis of fatty acid synthase complex (FAS), acyl is transferred to acyl carrier protein (ACP) in the order of two carbons per cycle. In this process, the fatty acid chain is extended. In the FAS complex, β-ketoacyl-ACP synthases (KASs) catalyze the condensation reaction of fatty acid chain extension. Finally, acyl-ACP is hydrolyzed by two types of acyl-ACP thioesterase (FATA and FATB) to release the acyl chain and form fatty acid. The unsaturated fatty acids are formed by fatty acid desaturases (FADs) inserting double bonds at specific positions of fatty acid chains [31]. Fatty acid elongation occurs in the endoplasmic reticulum [32]. The long-chain fatty acids and their derivatives form wax and cutin on the surface of plant leaves [18]. In this study, acyl-ACP thioesterase genes *FATA* and *FATB* were found to be key genes in the fatty acid biosynthesis under low-temperature treatment in FG and FX, respectively. It has been reported that FATA and FATB enzymes prefer unsaturated and saturated substrates, respectively. FATA enzyme preferentially catalyzes the hydrolysis of C18:1-ACP, while FATB enzyme catalyzes the hydrolysis of C16:0/C18:0-ACP [33,34]. This suggests that it is more inclined to form unsaturated fatty acids in FG and saturated fatty acids were formed in FX under low-temperature treatment.

The roles of fatty acids in plant stress have been reported in many studies, especially the role of unsaturated fatty acids in plant low-temperature stress. The fatty acid desaturase genes contribute a lot to plant cold tolerance by modulating the unsaturation of fatty acids. Overexpression studies of these genes have been performed in many species like tobacco [35], rice [13] and tomato [36], and have been proved to enhance cold tolerance in plants. In the present study, we found that desaturase genes *FAD3* and *FAD5* contributed to fatty acid synthesis in FG, and *FAD5* in FX. Therefore, more research on these two genes should be carried out in *Z. bungeanum* under low-temperature stress to further explore their functions. Particularly, we found that *SLD2* played an important role in the low-temperature response of two varieties of *Z. bungeanum*. *SLD2* is a sphingolipid long-chain base (LCB) delta 8 desaturase gene. Sphingolipids are the main lipid components of plant cell intimal systems, and play an important role in cell membrane structure and signal transduction [37]. The LCB is a unique component of sphingolipids [38]. The two double bonds in LCB are mostly introduced by *SLD* gene, which has been reported to alter the response of *Arabidopsis* mutants to long-term exposure to low temperature [39]. Therefore, the *SLD2* gene found in this study plays an important role in the sphingolipid components of *Z. bungeanum* under low temperature, and responds to cold. In addition, the very long-chain fatty acid cutin and wax on the plant surface are the first protective barrier of the plant defense system [18]. Wax is a mixture of a series of hydrophobic compounds, the main components of which are long-chain fatty acids and their derivatives [40]. A study has shown that the accumulation of wax can alleviate the water loss of *Arabidopsis* leaves under low-temperature stress [41]. Wax synthesis is a very complex process in epidermal cell, which requires the involvement of various enzymes. In this study, we found that *CER3,* a key gene in fatty acid synthesis and metabolism in FG under low-temperature stress, is related to the synthesis of aldehydes and alkanes [41,42]. Ni [19] reported that expression of *CER3* in Arabidopsis was down-regulated under low-temperature stress. The results in our study are contrary to this result, indicating that for *CER3,* the modes of action vary in different species. At present, the interaction between wax and low temperatures in *Z. bungeanum* and the role of *CER* genes are still unclear. Therefore, the function of *CER3* gene under cold stress needs to be further studied, which will involve study of the effects of wax in *Z. bungeanum* under low temperature. The key fatty acid synthesis-related genes identified in this study can be used as candidate genes for improving cold resistance through molecular breeding in *Z. bungeanum*.

In addition, fatty acid-related gene modules were constructed by WGCNA, among which MEfirebrick4 and MElightpink3 modules were highly correlated with the main fatty acid compositions in FG and FX. In the two modules, we found that numerous genes were enriched in plant hormone signal transduction pathway. This suggests that there is cross-talk between fatty acid and hormone responses to low temperature in *Z. bungeanum*. The results of the study by Wang et al. [22] confirmed this speculation. The mechanism of fatty acid and hormone interactions in *Z. bungeanum* needs to be explored by conducting more experiments. In a recent study, the target gene of MYB88 transcription factor in apple, the circadian clock-related gene *MdTIC*, was found to regulate plant cold tolerance by altering the saturation of fatty acids [24]. In our results, plant circadian rhythm-related genes were also identified, as well as some transcription factors. These genes provide candidates for further studies on low-temperature response mechanisms in *Z. bungeanum*. Therefore, the gene network constructed by WGCNA can provide a reference for further in-depth studies on *Z. bungeanum* cold response.

## 4. Materials and Methods

### 4.1. Plant Materials and Cold Treatment

The cold-tolerant and sensitive *Z. bungeanum* varieties ‘Fuguhuajiao’ (FG) and ‘Fengxiandahongpao’ (FX) were selected for this study. The variety FG grows in areas with higher latitude and lower temperature (38°42′ N~39°35′ N, 110°22′ E~111°14′ E, mean temperature 9.1 °C). The leaf wax layer of FG is thicker. Additionally, it has the growth characteristics of late flowering and strong stress resistance. The variety FX grows in areas with relatively high temperature (33°34′ N~34°18′ N, 106°24′ E~107°7′ E, mean temperature 12.1 °C). FX plants germinate earlier in the spring and are sensitive to low temperature. FX is widely cultivated because of its excellent fruit quality, including its rich aroma and bright color. The seeds of two varieties were harvested in the field at the Research Centre for Engineering and Technology of *Zanthoxylum*, State Forestry Administration, Northwest A&F University, Fengxian, Shaanxi Province, China. The seeds of *Z. bungeanum* were sowed into the burrow plate for germination after degreasing and soaking in warm water, and then transferred to flowerpots. Seedlings were grown in a greenhouse (25–28 °C, 16/8 h day/night) until 4 months old. Leaves were collected at 0, 1, 3, 6, 12, and 24 h after 4 °C cold-stress treatment (9 seedlings comprised a biological replicate, with a total of three replicates), frozen in liquid nitrogen immediately, and stored at −80 °C for further determination and analysis. ‘Fuguhuajiao’ and ‘Fengxiandahongpao’ were marked as FG (FG1–18, 6 time points × 3 biological replicates) and FX (FX1–18, 6 time points × 3 biological replicates).

### 4.2. Fatty Acid Extraction and Composition Analysis by Gas Chromatography–Mass Spectrometry (GC-MS)

The fatty acids in leaves (0.5 g of each sample) were extracted by 3 mL mixed solvent (chloroform: methanol = 2:1). A total of 3 extractions were carried out. The concentrated extract (2 mL) was transferred to a transparent glass bottle, and 5 mL methanol solution with 1% sulphuric acid was added for methyl esterification of fatty acid. Methyl esters of fatty acids were extracted by 2 mL n-hexane. The fatty acid component analysis was performed by Thermo Scientific Trace 1310 gas chromatography (Thermo Fisher Scientific Inc., Waltham, MA, USA) with a flame ionization detector (FID).

Gas chromatographic conditions: the carrier gas was helium gas with a purity of 99.99%, the carrier flow was 1 mL/min, and the split flow was 20 mL/min. Temperature program: the initial temperature was 80 °C for 1 min, and was then increased to 175 °C with a rate of 50 °C/min for 1 min. Then the temperature was increased to 200 °C with a rate of 5 °C/min and maintained for 1 min, and further increased to 210 °C with a rate of 2 °C/min and maintained for 1 min. In the last step, the temperature was increased to 230 °C with a rate of 5 °C/min and maintained for 15 min. MS condition: the ion source temperature was 280 °C, the transfer line temperature was 240 °C. The qualitative and quantitative analysis of fatty acids was performed using external standard method by employing authentic methyl esters mixture standards (C4–C24, Shanghai yuanye Bio-Technology Co., Ltd., Shanghai, China).

### 4.3. RNA Extraction and Sequencing

Total RNA was extracted from each sample using the RNAprep pure plant kit (Tiangen Biotech, Beijing, China) according to the manufacturer’s instructions. The quality, integrity and quantity of the extracted RNA were detected using a NanoDrop 2000 spectrophotometer (Thermo Fisher Scientific, Wilmington, DE, USA) and an Agilent Bioanalyzer 2100 system (Agilent Technologies, Palo Alto, CA, USA). Transcriptome sequencing was performed on the Illumina HiSeq 2500 platform (Illumina Inc., San Diego, CA, USA). HISAT2 [43] and StringTie [44] were used to perform the mapping and assembling work with the reference genome of Chinese prickly ash [45]. The sequencing data were available in the NCBI SRA database under accession number PRJNA597398.

### 4.4. Quantitative Real-Time PCR Analysis of Fatty Acid Related Genes

Twenty genes were selected to be determined by qRT-PCR for expression level analysis. All the fatty acid metabolism pathway-related genes in this study were selected based on RNA-seq data. First-stand cDNA synthesis was carried out using PrimeScript RT Master Mix (TaKaRa, Dalian, China). qRT-PCR was performed using 2× Sybr Green qPCR Mix (BIOV, Beijing, China) and run on a StepOnePlus Real-time PCR System (Thermo Fisher Scientific, Wilmington, DE, USA). Reference genes *ACTIN* and *UBQ* were applied as internal controls. Primers were presented in Table S1. Relative expression levels were calculated according to the 2^−^^△△CT^ method [46].

### 4.5. Data Analysis

OriginPro 2017C (Originlab, Northampton, MA, USA) was used for data standardization and principal component analysis (PCA). Cluster heat map (CHM) analysis was carried out by TBtools [47]. Orthogonal partial least squares discriminant analysis (OPLS-DA) was carried out using online software (Available online: https://www.omicshare.com/tools/Home/Soft/getsoft (accessed on 25 January 2021)). Redundancy analysis (RDA) was performed by Canoco 5 program. The weighted gene co-expression network (WGCNA) was analyzed by R package [48]. The transcriptome data used for WGCNA were from our previous work [49]. The co-expression network was visualized by using Cytoscape 3.7.1 [50].

## 5. Conclusions

The present work had the main aim of investigating the variation in fatty acid profiling in *Z. bungeanum* under low-temperature treatment. Twelve main fatty acids were detected in the leaves of two *Z. bungeanum* varieties: isomyristic acid, palmitic acid, palmitoleic acid, isostearic acid, stearic acid, oleic acid, linoleic acid, linolenic acid, eicosanoic acid, eicosenoic acid, cetoleic acid and lignoceric acid. Palmitic acid and linolenic acid were dominant in both *Z. bungeanum* varieties. More unsaturated fatty acids were synthesized in FG under cold stress. Low temperature affected the compositions and contents of fatty acids in *Z. bungeanum*, and they varied in the two varieties. OPLS-DA results showed that the important fatty acids in FG and FX were inconsistent. Analysis of molecular levels revealed that FG preferred to synthesize unsaturated fatty acids, while FX preferred to synthesize saturated fatty acids. Fatty acid synthesis pathway genes *FATA*, *SLD2*, *CER3, FAD3* and *FAD5* were identified as the key genes affecting the fatty acid composition in cold-tolerant variety FG, while *SLD2*, *KAS I*, *FATB, FAD5* and *ACC* were key genes in cold-sensitive variety FX under low-temperature stress. The above results showed that the variation and strategies of fatty acids in two varieties of *Z. bungeanum* were different under low-temperature stress. This work provides a reference for the investigation of chemical components in *Z. bungeanum* in response to environmental stresses.

## Figures and Tables

**Table 1:** Indexes of fatty acid composition in FG and FX leaf samples under 4 °C low-temperature stress for 24 h.

Fatty Acid (mg·g^−1^)	TFA	USFA	SFA	IUFA
FG-0 h	1.92 ± 0.10 ^cde^	1.14 ± 0.11 ^cde^	0.78 ± 0.03 ^b^	2.63 ± 0.19 ^cd^
FG-1 h	2.52 ± 0.20 ^bc^	1.63 ± 0.14 ^b^	0.89 ± 0.06 ^b^	4.30 ± 0.38 ^b^
FG-3 h	2.41 ± 0.27 ^bcd^	1.57 ± 0.18 ^bc^	0.84 ± 0.09 ^b^	4.13 ± 0.47 ^b^
FG-6 h	2.32 ± 0.24 ^cde^	1.51 ± 0.17 ^bc^	0.81 ± 0.07 ^b^	3.99 ± 0.47 ^b^
FG-12 h	2.51 ± 0.14 ^bc^	1.68 ± 0.08 ^b^	0.84 ± 0.06 ^b^	4.50 ±0.22 ^b^
FG-24 h	4.11 ± 0.99 ^a^	2.93 ± 0.72 ^a^	1.18 ± 0.27 ^a^	8.03 ± 1.96 ^a^
FX-0 h	1.72 ± 0.24 ^e^	0.98 ± 0.16 ^de^	0.74 ± 0.08 ^b^	2.38 ± 0.36 ^cd^
FX-1 h	1.91 ± 0.18 ^cde^	1.04 ± 0.10 ^de^	0.88 ± 0.08 ^b^	2.64 ± 0.25 ^cd^
FX-3 h	1.67 ± 0.12 ^e^	0.85 ± 0.05 ^e^	0.81 ± 0.08 ^b^	2.13 ± 0.11 ^d^
FX-6 h	1.83 ± 0.17 ^de^	0.91 ± 0.09 ^de^	0.93 ± 0.09 ^b^	2.16 ± 0.19 ^d^
FX-12 h	3.02 ± 0.38 ^b^	1.81 ± 0.29 ^b^	1.21 ± 0.10 ^a^	4.67 ± 0.76 ^b^
FX-24 h	2.24 ± 0.09 ^cde^	1.34 ± 0.09 ^bcd^	0.90 ± 0.02 ^b^	3.52 ± 0.28 ^bc^

Values are displayed as means ± standard deviation. The superscript letters show the statistical differences in significant level at 5%. Indicators with different letters represent significant differences between them. TFA: total fatty acids; USFA: unsaturated fatty acids; SFA: saturated fatty acids; IUFA: the index of unsaturated fatty acids.

## Supplementary Materials

The following supporting information can be downloaded at: https://www.mdpi.com/article/10.3390/ijms23042319/s1.
