# Supplementary figures and images for: Low Temperature Affects Fatty Acids Profiling and Key Synthesis Genes Expression Patterns in Zanthoxylum bungeanum Maxim
Source: Int J Mol Sci. 2022 Feb 19;23(4):2319. doi: 10.3390/ijms23042319 (PMC8876529; doi:10.3390/ijms23042319)

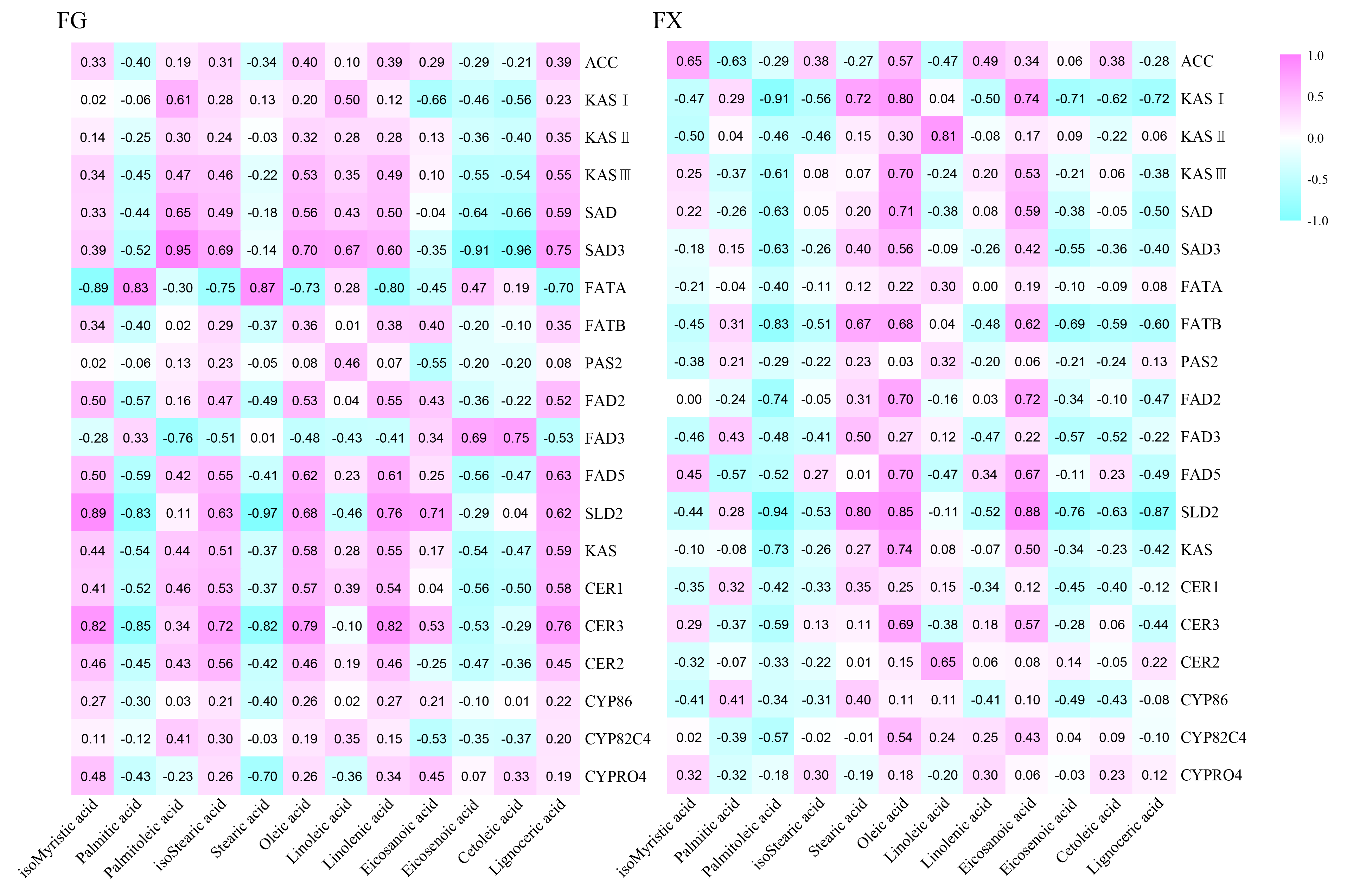

Supplement: Supplementary file 1 [file ijms-23-02319-s001.zip › FigureS1.tif]
